# A Comparison of Single Dimension and Volume Measurements in the Risk Stratification of Pancreatic Cystic Lesions

**DOI:** 10.3390/jcm12185871

**Published:** 2023-09-09

**Authors:** Da Yeon Ryoo, Bryn Koehler, Jennifer Rath, Zarine K. Shah, Wei Chen, Ashwini K. Esnakula, Phil A. Hart, Somashekar G. Krishna

**Affiliations:** 1 Department of Internal Medicine, Ohio State University Wexner Medical Center, Columbus, OH 43210, USA; dayeon.ryoo@osumc.edu (D.Y.R.); bryn.koehler@osumc.edu (B.K.); 2 Department of Radiology, Ohio State University Wexner Medical Center, Columbus, OH 43210, USA; jennifer.rath@osumc.edu (J.R.); zarine.shah@osumc.edu (Z.K.S.); 3 Department of Pathology, Ohio State University Wexner Medical Center, Columbus, OH 43210, USA; wei.chen@osumc.edu (W.C.); ashwini.esnakula@osumc.edu (A.K.E.); 4 Division of Gastroenterology, Department of Internal Medicine, Ohio State University Wexner Medical Center, Columbus, OH 43210, USA; philip.hart@osumc.edu

**Keywords:** pancreatic cancer, pancreatic cystic lesion, IPMN, CT, MRI, MRCP, EUS, maximum diameter, volume

## Abstract

The incidence of pancreatic cystic lesions (PCLs) has been rising due to improvements in imaging. Of these, intraductal papillary mucinous neoplasms (IPMNs) are the most common and are thought to contribute to almost 20% of pancreatic adenocarcinomas. All major society guidelines for the management of IPMNs use size defined by maximum diameter as the primary determinant of whether surveillance or surgical resection is recommended. However, there is no consensus on how these measurements should be obtained or whether a single imaging modality is superior. Furthermore, the largest diameter may fail to capture the complexity of PCLs, as most are not perfectly spherical. This article reviews current PCL measurement techniques in CT, MRI, and EUS and posits volume as a possible alternative to the largest diameter.

## 1. Introduction

Despite recent advances in cancer detection and management strategies, pancreatic cancer remains among the deadliest worldwide. In the United States, pancreatic cancer is responsible for 8.2% of all cancer deaths and has an estimated 5-year survival of 11.5 percent [1]. The global incidence is projected to increase to 18.6 per 100,000 by the year 2050, which poses a significant public health burden [2]. There are numerous well-established risk factors for developing pancreatic cancer, including cigarette smoking, family history, age, and male sex. However, due to the lack of effective screening methods, the only populations recommended to undergo any type of screening are those with a significant family history or those with a high-risk genetic screen. For this reason, the effective management of premalignant pancreatic lesions is crucial in both slowing the incidence of cases and improving mortality. 

Pancreatic cystic lesions (PCLs) represent a significant portion of premalignant pancreatic lesions and contribute to nearly 20% of pancreatic adenocarcinomas [3]. PCLs are classified as mucinous and non-mucinous. Mucinous PCLs, which include intraductal papillary mucinous neoplasms (IPMNs) and mucinous cystic neoplasms (MCNs), have the potential to progress to pancreatic adenocarcinoma. The surveillance of mucinous PCLs poses a promising opportunity to advance the detection and prevention of pancreatic cancer. Improvements in cross-sectional imaging and its increased utilization in an aging population have contributed to a marked increase in the incidence of PCLs [4]. Due to the heterogeneous nature of these lesions, the challenge lies in accurately classifying them in order to guide management. 

Among neoplastic PCLs, IPMNs are the most common. The point prevalence of IPMNs in those above age 60 is ~1 in 100 subjects [5,6]. While many IPMNs are discovered incidentally and are asymptomatic, rarely, they can cause symptoms, including abdominal pain, pancreatitis, and jaundice. They also have a significant malignant potential, with a mean malignancy rate of 61% in main-duct IPMNs (MD-IPMNs) when the duct diameter is ≥10 mm and a malignancy rate of 25.5% in branch-duct IPMNs (BD-IPMNs) when the cyst diameter is ≥4 cm [7]. Specifically, the risk of malignant transformation of BD-IPMNs is 18–25% at 3–4 cm and >25% at ≥4 cm diameter [8,9]. 

The early detection of high-risk BD-IPMNs is crucial, as the 5-year survival after the surgical resection of IPMNs with invasive cancers ranges from 31 to 60%, compared to 90 to 100% in noninvasive IPMNs [10]. Unfortunately, there are significant limitations in the imaging methods used to identify and risk-stratify BD-IPMNs. Guidelines from multiple professional societies use PCL size to assess the risk of malignant transformation to determine the appropriate surveillance or intervention [9,11,12,13,14,15,16]. However, these guidelines are difficult to apply since various imaging modalities, including computed tomography (CT) scan, magnetic resonance imaging (MRI)/magnetic resonance cholangiopancreatography (MRCP), and endoscopic ultrasound (EUS), often provide discordant measurements. Therefore, consensus regarding optimal imaging modality and best practices in collecting measurements is needed to ensure that BD-IPMNs are appropriately managed. 

## 2. Current Guidelines Regarding Pancreatic Cystic Lesion Measurement

There are five sets of guidelines from major societies regarding the management of PCLs. Each set of guidelines seeks to establish high-risk features summarized in Table 1 that should prompt closer follow-up or surgical evaluation. While these features vary, PCL size is the one factor uniformly cited by all guidelines. The American College of Gastroenterology (ACG), the American College of Radiology (ACR), the American Gastrointestinal Association (AGA), and the International Association of Pancreatology (IAP) established a diameter of at least 3 cm as indicative of increased risk for advanced neoplasia, whereas the European evidence-based guidelines use a cutoff of 4 cm in diameter. Some of these guidelines, specifically the European and IAP/Fukuoka guidelines, provide management recommendations for specific types of PCLs, whereas the ACG and AGA guidelines offer general strategies for PCL surveillance and management regardless of subtype. The revised 2017 IAP/Fukuoka guidelines are commonly used as they provide specific operative criteria for MD- and BD-IMPNs. According to IAP/Fukuoka guidelines, high-risk stigmata are defined as obstructive jaundice, main pancreatic duct >10 mm, and enhancing mural nodule >5 mm. Surgical resection is strongly recommended for both MD- and BD-IPMNs with any high-risk stigmata. Size-guided surveillance imaging is recommended for BD-IPMNs that are <3 cm and without other worrisome or high-risk features [9]. 

Despite the consistent use of PCL size as one of the primary determinants of surveillance and management recommendations for BD-IPMNs, there is inconsistent evidence to support this. The two major considerations in cyst measurement are baseline size and incremental growth, where a rate of growth greater than 5 mm every 2 years is regarded as a worrisome feature [9]. However, the consistent implementation of these recommendations is impaired by insufficient consensus on imaging protocols and measurement techniques. The ACR guidelines are the only ones to specifically discuss CT and MRI protocols. Therefore, this article seeks to clarify how PCL measurements are obtained across imaging modalities and determine whether there are discrepancies to be considered in establishing management guidelines. 

## 3. Methods of Measuring PCL Size

### 3.1. CT, MRI, and EUS

As noted in most of the PCL management guidelines, the three most common imaging modalities for detecting and diagnosing pancreatic cysts are CT, MRI, and EUS. Each modality comes with its own risks and benefits. While MRI can provide the high contrast definition of soft tissue without exposing patients to radiation, it is more time-consuming and expensive than CT [17]. CT is more widely available and time efficient, but it exposes patients to repeated doses of radiation throughout the pancreatic cyst surveillance period. EUS provides the high-resolution imaging of pancreatic cysts with the option to utilize fine needle aspiration (FNA) during the procedure to further assist with the diagnosis of the cyst. However, it is by far the most invasive method of the three [17]. In this review, several studies that measured PCLs were assessed to investigate the differences in imaging modality and size measurement methodology. To account for recent advances in imaging technologies, only studies conducted within the past 15 years were reviewed.

### 3.2. Maximum Diameter—Variation between Imaging Modalities 

Du et al. compared the difference in cyst size measurement and cyst characteristic appearance across CT, MRI, and EUS; (*n* = 68). They used cyst diameter as the definition of cyst size. While there were no major discrepancies in size, EUS was superior in identifying specific characteristics, such as intracystic nodules, wall thickness, and septations [18]. Similarly, Boos et al. compared incidental pancreatic cyst size measurement (defined by maximal diameter) between CT and MRI and reported a mean absolute size discrepancy of 2.1 ± 1.8 mm (*n* = 267; median 1.5 mm, range 0–9 mm). This study also determined that the larger the cyst size, the larger the absolute size discrepancy between the imaging modalities [19]. Moreover, CT did not correctly identify incidental PCLs by rate of 22% when compared to MRI. 

### 3.3. Maximum Diameter—Imaging vs. Histopathology

Lee et al. assessed the PCLs of patients who had all CT, MRI, and EUS images taken within three months prior to surgical resection (*n* = 34). The authors measured the maximum dimension of the pancreatic cyst in two axes—cross-sectional and coronal—in all three imaging modalities. The larger of the two measurements was selected to define cyst size in the analysis. Of the three imaging modalities, EUS had the widest range of 95% limits of agreement (−17.43 to +23.87) and very good reliability with an intraclass correlation coefficient of 0.84 (95% CI 0.58–0.94) for mucinous lesions. EUS was specifically found to underestimate the size of PCLs located in the pancreatic tail when compared to CT and MRI [20]. The authors concluded that EUS findings should be interpreted with caution, particularly when the lesion is located in the tail of the pancreas and is relatively large in size.

Maimone et al. compared the cyst size measurements of 175 patients who underwent some combination of CT, MRI, and EUS imaging prior to surgical resection. They defined cyst size as the single largest cyst diameter. The median size differences between each combination of imaging modalities were: 4 mm (0–25 mm) between EUS and CT, 4 mm (0–17 mm) between EUS and MRI, and 3 mm (2–20 mm) between CT and MRI. Histopathologic data from resection were then compared to 12 EUS, 13 CT, and 8 MRI measurements. The median size differences were: 9.5 mm (0–20 mm) between EUS and pathology, 5 mm (0–21 mm) between CT and pathology, and 5.5 mm (2–44 mm) between MRI and pathology [21]. In this study, the authors noted that there was significant variation in the size estimates of PCLs when assessed using different imaging modalities. Therefore, they recommended the use of a single imaging modality for surveillance to ensure consistency in size measurements.

Two additional studies also compared PCL size data obtained from imaging to surgically resected pathology specimens. Leeds et al. compared the maximum diameters of cysts measured with CT and EUS to the measurements of surgically resected pathology specimens in 70 patients. Measurements included maximum diameter in the axial, sagittal, and coronal planes. There were no significant differences found between measurements obtained via either imaging modality when compared to pathology [22]. Huynh et al. similarly used a small sample size of 57 IPMNs (3 MD, 41 BD, 13 mixed) to compare the three imaging modalities on the strength of their size measurement correlation to the pathological cyst size. Each of the three imaging modalities was used to measure the maximum long-axis cyst diameter, which was later compared to the post-operative pathological cyst maximum diameter. Unlike Leeds et al., this study revealed that CT and MRI significantly overestimated the IPMN size measurement when compared to pathological cyst measurement, while EUS best predicted the pathological cyst size, especially for those smaller than 3 cm [23]. The authors speculated that the differences in cyst size measurement across different imaging modalities could be attributed to the difference in cyst size in coronal and axial views of CT and MRI as opposed to oblique angle views on EUS. Aside from investigating their hypothesis, the authors additionally commented on the lack of standardized protocol in radiographic and pathological pancreatic cyst size measurement.

### 3.4. Diameter and Volume Estimation—Imaging vs. Histopathology

**Figure 1:**
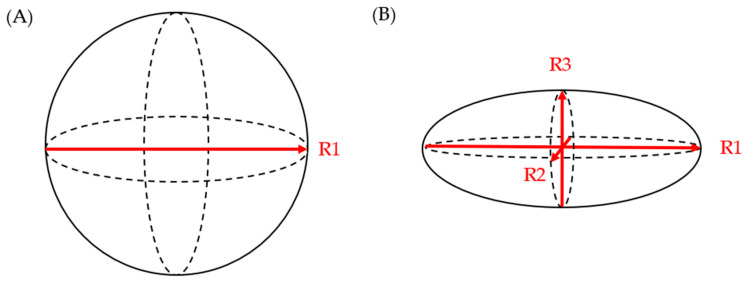
(**A**) spherical volume = π × R1^3^/6; (**B**) ellipsoid volume = R1 × R2 × R3 × π/6.

Literature evidence from the last decade suggests that the three-dimensional growth pattern of PCLs is uneven and may not be accurately estimated when only one or two dimensions are obtained in imaging studies [24]. Chalian et al. compared PCL volumes obtained from CT imaging to the volume of fluid aspirated during EUS. The CT measurement of cyst volume was measured using (a) software-assisted CT volumetry and (b) spherical and ellipsoid volume calculation formulas (spherical volume = π × R1^3^/6; ellipsoid volume = R1 × R2 × R3 × π/6 (R1: longest diameter on axial plane; R2: longest diameter on coronal plane; R3: longest diameter on sagittal plane)) (Figure 1). Whether a cyst was spherical or ellipsoid was determined using an elongation value, 1 − aspect ratio, or 1 − (width/length), where cysts that are spherical had an elongation value closer to 0 and those that were ellipsoid had an elongation value closer to 1. Of the 14 fully aspirated PCLs, the mean aspirated cyst volume was 2.05 ± 1.56 mL. The mean volume measured via CT volumetry was 2.27 ± 1.54 mL, while the ellipsoid volume (formula) yielded a mean volume of 2.94 ± 2.06 mL, and the spherical volume (formula) resulted in a mean volume of 3.78 ± 2.47 mL. Although software-assisted CT volumetry was the most accurate method, the utilization of the ellipsoid volume (formulas) was found to be preferable over the spherical volume (formulas) [25].

## 4. Prediction of Advanced Neoplasia in IPMNs—Variation between Imaging Modalities Using Consensus Criteria 

Multiple studies have compared the diagnostic accuracy of various imaging modalities (CT, MRI, and EUS) using the IAP/Fukuoka guidelines in predicting advanced neoplasia in IPMNs. In a study of 86 patients with IPMNs, the diagnostic performance of CT and MRI for the prediction of malignant IPMNs was comparable with good inter-modality agreement (*p* = 0.43; κ = 0.70) [26]. A meta-analysis of 28 studies encompassing 1812 patients with IPMN was conducted to further compare the diagnostic accuracy of multiple imaging modalities. Of the imaging modalities included in the study, PET/CT and MRI/MRCP had the highest overall diagnostic accuracy (area under the curve of summary receiver-operating characteristic curves of 0.92 and 0.87, respectively) and therefore supported the use of either imaging modality interchangeably when assessing the malignant potential of IPMNs [27]. A meta-analysis investigating the difference in diagnostic accuracy for diagnosing malignant PCLs between the three imaging modalities found MRI and CT to have comparable accuracy (sensitivity *p* = 0.822; specificity *p* = 0.096), while EUS showed lesser specificity compared to MRI (75% vs. 80%, *p* < 0.05). This study therefore posits that MRI may be a better imaging tool to guide PCL management than EUS [28].

## 5. Cyst Size as a Predictor of Advanced Neoplasia

As cited in Table 1, the available guidelines universally cite cyst size ≥ 3 cm as a worrisome feature for advanced pathology and therefore use size as a guide to surveil IPMNs. However, a large study evaluating BD-IPMNs (n = 2258; 36.7% with advanced neoplasia) showed that cyst size (OR 1.024, 95% CI 1.018–1.030), although predictive of advanced neoplasia, had lower odds ratios than main pancreatic duct (MPD) dilation, the presence of mural nodules, and elevation in CA 19-9. However, this study did not specify how the cyst size was defined and measured [29]. The association between cyst size and advanced neoplasia has been corroborated by multiple meta-analyses which also investigated the relationship between various BD-IPMN characteristics and their correlation with malignancy. Again, while cyst size ≥ 3 cm was associated with high-grade dysplasia and malignancy, the odds ratios were significantly lower than those of many other cyst characteristics [30,31]. A retrospective study of 269 patients who underwent the surgical resection of asymptomatic pancreatic cysts showed a remarkable discrepancy between the pathological diagnosis of advanced neoplasia and those expected to be at high risk for neoplasia based on the available guidelines. Of the 269 PCLs, 41 were found to have advanced neoplasia. Of these 41 patients, only 3 met the criteria for resection per AGA guidelines, 22 met the criteria per ACR guidelines, and 30 met the criteria per IAP/Fukuoka guidelines. These findings suggest a lower sensitivity of these guidelines in diagnosing advanced neoplasia. These guidelines were similarly inaccurate for low-grade or benign lesions. Of the 228 patients with low-grade or benign cysts, 27 would have met the criteria for resection per AGA guidelines, 89 for ACR guidelines, and 123 for IAP/Fukuoka guidelines. This correlated to an overall diagnostic accuracy of 49.8% for IAP/Fukuoka, 59.8% for ACR, and 75.8% for AGA guidelines [32]. Yet another study compared one center’s resection criteria—symptomatic, suspicious morphologic features through radiography and mucinous characteristics through fluid aspiration—to the size criteria of ≥3 cm used by most guidelines. Of the mucinous or cancerous lesions, 73% met the institution’s criteria but only 50% were ≥3 cm [33].

This inconsistent evidence has prompted some investigators to question whether other criteria might better predict the malignant risk of BD-IPMNs than cyst size. The growth rate has been proposed as a possible alternative. A study of 52 patients with BD-IPMN diagnosed by ERCP or MRI/MRCP had a mean follow-up of 31.2 months to assess for changes in maximum diameter and MPD diameter. Seven of the lesions demonstrated growth on follow-up imaging, and both cyst size > 3 cm and MPD dilatation were associated with an increased likelihood of growth [34]. El Chafic et al. queried whether growth rate may be superior to size at baseline in predicting advanced neoplasia. However, an analysis of 161 patients with BD-IPMN demonstrated that rapid growth, defined as a mean growth rate percentage ≥ 30% per year, was not associated with advanced neoplasia on surgical pathology and did not correlate with other high-risk patient characteristics [35]. Conversely, Ciprani et al. found that, for small PCLs < 15 mm (n = 816), the strongest predictor of malignancy was a growth rate ≥ 2.5 mm per year [36]. 

Recently, some investigators have evaluated cyst volume as an alternative predictor of malignant risk, as it is proposed to be more accurate for lesions that are not perfectly spherical. For example, a recent radiology guideline paper proposed volumetry as a more objective alternative to diametric size [37]. Studies comparing imaging modalities in other cancers have shown that a small level of growth in the diameter of lesions is associated with a much larger increase in cyst volume. This is true for even perfectly spherical lesions, where a growth of 20% in diameter correlates with a volume increase of 72.8% [38]. Furthermore, volume growth has been shown to be significantly higher in patients who developed worrisome PCL features [39]. 

## 6. Cyst Volume Assessment in Other Organs

Multiple imaging modalities, including ultrasound, CT, and MRI, are already being used to assess volume and risk stratify lesions in other solid organs. There is a well-established precedent for this in solid masses. Buerke et al. demonstrated the feasibility and precision of volume assessment of peripheral, abdominal, and thoracic lymph nodes using CT imaging [40]. Subsequently, in a study of primary lung cancers, three reviewers assessed 64 lung tumors on CT scans to compare diametric, areametric, and volumetric measurements. While all three measures had high reproducibility, volumetric measurements were more precise than traditional diametric ones [41]. The accuracy of MRI for solid tumor volume assessment was reinforced by the study of male patients with elevated PSA who underwent radical prostatectomy. MRI tumor volumes were obtained via manual tumor segmentation and compared to histopathologic tumor volumes. Accurate estimates of histopathologic volume were obtained using MRI volumetry, and the accuracy was greater for tumors larger than 0.5 cm^3^ [42]. 

Despite the precedent of solid lesion volumetry with CT and MRI, image-guided volumetry is less frequently used in cystic lesions. Autosomal dominant polycystic kidney disease (ADPKD) is an example where cystic lesion volumes are routinely measured using imaging [43]. Multiple techniques are utilized to obtain total kidney and individual cyst volumes to evaluate disease progression. These range from manual planimetry to semi- or even fully-automated techniques [44]. Manual planimetry has historically been considered the gold standard for ADPKD cyst volume evaluation but is very time-consuming. It requires a reviewer to manually trace the outline of a lesion, calculate the total volume by multiplying all traced areas by axial slice thickness, and then combine slice volumes [44]. Another method of image-guided volume measurement used in ADPKD management is stereology. Areas corresponding to kidney regions are defined with grid points in serial coronal sections of MRI. The areas of cysts or renal parenchyma are then calculated by counting the number of intersections within them and converting this into a pixel count. The renal or cystic volume is then calculated by summing the products of the resulting areas and corresponding slice thickness [44]. 

The ellipsoid volume formula method (V = length (average of sagittal and coronal lengths) × width × depth × (π/6)) uses the measurements of longitudinal length, maximum width, and maximum depth from coronal and sagittal MRI slices to calculate volume [45]. The mid-slice method uses a manual tracing on a single middle coronal slice of MRI to calculate area, which is then multiplied by total number of slices, slice thickness, and experimentally defined correction factor to calculate volume [45]. In semi-automated ADPKD cystic volume measurement technique, an algorithm is used to generate a contour using a manually selected reference point in a central slice on MRI [44]. Both CT and MRI have been shown to reliably estimate total kidney and individual cyst volumes in patients with ADPKD [46,47]. These volumes are important for understanding the disease course, as high cyst volumes are negatively associated with renal function, and those with high ratios of cyst volume to total kidney volume have a higher likelihood of requiring dialysis for end-stage renal disease [47,48]. Similar techniques are now being employed in the evaluation of pancreatic cystic lesions.

## 7. Methods of Volume Assessment for Managing PCLs

Given the lack of evidence that PCL size by the maximum diameter is a reliable tool for determining malignant risk, recent research has focused on PCL volume as a possible alternative for risk assessment. Awe et al. reviewed 195 patients with PCLs at a single center to correlate size, as measured by maximum axial diameter (MAD), and volume. For MAD measurement, a region of interest (ROI) was drawn in the axial dimension of the CT imaging slice with the largest MAD. Then, a quantitative imaging software platform was used to generate a 3D ROI and edited to exclude vasculature, ducts, surgical hardware, and bowel gas. After a final 3D ROI was generated, the software generated values for MAD, volume, surface area, and sphericity. Results showed that MAD is a poor correlate of volume in smaller cysts (1–3 cm). When there were subsequent CT or MRI images collected over a year later for comparison, MAD changes over that time also correlated poorly with volume changes. Unsurprisingly, these estimates were even less reliable in non-spherical cysts. Therefore, the authors concluded that MAD incompletely captures the complexity of pancreatic cysts [49]. Notably, this study was limited by a lack of pathologic data to correlate cyst characteristics with malignant risk, as most of the patients underwent surveillance without surgical resection. 

Similarly, a single-center retrospective study by Pandey et al. evaluated 164 IPMNs on 107 MRI images and compared manual and semi-automatic largest diameter and volume measurements between three radiologists to assess for interobserver reproducibility. All three readers were taught a standard protocol to obtain these measurements. First, each reader measured the largest diameter of the IPMN manually using electronic calipers on both axial T2W and coronal three-dimensional MRCP images on the cross-section with the largest diameter. They then excluded any ductal extension and separated groups of cysts if there was a dividing septum >1 mm thick. This was followed by semi-automatic measurements, which involved the interactive segmentation of the lesion on each slice where the lesion was visible. All measurements were performed using a commonly available commercial Picture Archiving and Communication System (PACS; Carestream Health, Inc., Rochester, NY, USA) software. Of the six measurements collected for each IPMN, the highest interobserver reproducibility was seen for axial manual diameter measurements in cysts ≥1.5 cm, while the lowest was seen for coronal manual diameter measurements on cysts measuring <1.5 cm. This overall high interobserver reproducibility was attributed to the standardized measurement protocol taught to the three readers at the beginning of the study. Therefore, the authors concluded that a standardized cyst measurement technique would benefit IPMN follow-up since each follow-up image is often read by different radiologists. Additionally, the semi-automatic method of volume measurement in this study did not rely on the subjective selection of a cyst cross-section with the largest diameter by the reader, suggesting that cyst volume measurement may allow for more a reproducible cyst monitoring mechanism, especially in the absence of a standardized measurement protocol [24]. 

Pozzi Mucelli et al. assessed 106 patients with a histopathological diagnosis of BD- and mixed-type IPMN with an available preoperative MRI to test the hypothesis that volume could serve as a better predictor of malignancy than size. All MRIs were evaluated on a PACS by two radiologists in consensus reading, with one cyst chosen per patient (the largest or one with the highest suspicion for malignancy). Several IPMN parameters were collected, including a maximum diameter on axial and coronal T2W images, elongation value (defined as 1-(width/length)), maximum MPD diameter, the presence of contrast-enhancing mural nodules, cyst wall thickening ≥ 2 mm, growth of >5 mm per year during follow-up, solitary vs. multifocal, location, and volume. Volume was calculated on axial T2W images, on which an ROI was drawn along the edge of the BD-IPMN on multiple levels. The software then automatically calculated the volume. Interestingly, the analysis of these data showed that neither elongation value nor volume were associated with advanced neoplasia. This is despite the confirmation with elongation value data that most of these lesions were not spherical. The only variables in the study which were associated with advanced neoplasia were the presence of contrast-enhancing mural nodules, a diameter of MPD ≥ 5 mm, and serum CA 19-9 level > 37 [50]. 

## 8. Conclusions

**Figure 2:**
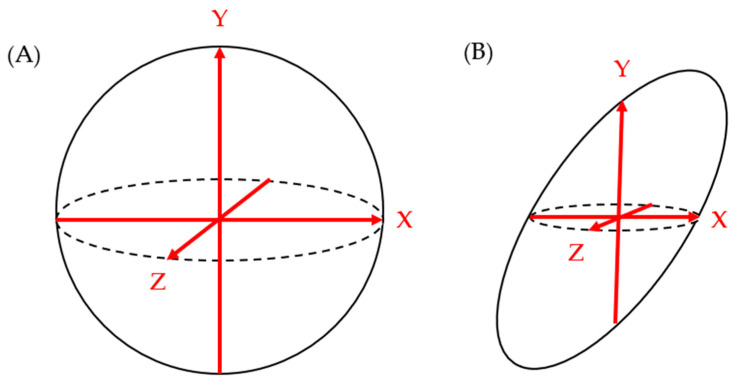
While the maximum length measurements on each of the X-Y-Z axes correlate well to overall spherical shape of figure (**A**), they do not correlate as well to the non-spherical shape of figure (**B**).

Studies that utilized PCL size as one of the topics of investigation often used the maximum diameter of cysts seen on a 2D imaging modality (MRI, CT, and/or EUS) to define the cyst size. This generalized sizing technique assumes that pancreatic cysts are roughly spherical and that the largest diameter captures the overall size of the cyst. However, this does not reflect the natural variability of PCLs, which can have a multitude of morphological appearances [51]. Therefore, the utilization of maximum diameter on 2D imaging has its shortcomings. Firstly, the maximum diameter depends heavily on the reader’s choice of the image plane (axial vs. sagittal vs. coronal) as well as the angle of the images relative to the lesion. Most conventional CT scans and MRIs provide three-plane imaging: coronal, sagittal, and axial. Even if the largest maximum diameter from each of the three views is chosen for the cyst size parameter, there is a chance of overestimating or underestimating the cyst size, depending on the cyst orientation (i.e., the axis of cysts’ actual maximal diameter may not be oriented parallel to the image plane) and shape, as shown in Figure 2. EUS offers more freedom in angle of view compared to CT and MRI with a vantage point closer to the cyst of question. However, the view is still limited by the probe’s angle of approach to the cyst relative to its orientation and shape. While the maximum cyst diameter has the benefit of being easily measured from any imaging modality, the drawback of this convenience is a lack of standardized protocol for obtaining the measurement. Even the ACG and European guidelines do not comment on the best practices for measuring pancreatic cyst size, despite using it as the primary criteria for the diagnosis, management, and surveillance of pancreatic cysts.

The limitations of the maximum diameter measurement on 2D imaging can be somewhat overcome by measuring the cyst’s length, height, and width to appropriately capture the three-dimensional volume the cyst occupies rather than merely its maximal length. Such measurements can more accurately evaluate a cyst that does not comply with the spherical assumption. However, the measurements are again restrained by the angle of view available in each imaging modality. The cyst may be oriented in such a way that three perpendicular views are inadequate to estimate an accurate cyst size. While three-axis measurement has the potential to offer improved accuracy by allowing for the calculation of volume, it may be more difficult and time-consuming to obtain and report. Several studies discussed above evaluated the efficacy and reliability of cyst volume measurements, not only in the pancreas but in other organs as well. While one study has suggested the reproducibility of cyst volume measurement using a semi-automatic volume measurement method, another study did not find a strong correlation between cyst volume and the risk of malignancy. Therefore, additional larger studies to evaluate the relationship between cyst volume and malignant risk would provide guidance as to whether developing a uniform system to assess cyst volume would be useful. Given that cyst volume measurement can be relatively reliably reproduced using commercially available PACS software, collecting more data on cyst volume measurement through meta-analysis may prove to be valuable. 

The cited references unanimously voice the lack of standardized protocol of cyst size measurement and raise it as a potential threat to accurately monitoring pancreatic cysts. A study has demonstrated that a standardized cyst size measurement method can be taught to radiographic readers with high reproducibility. Such an assessment should be taken into account by entities creating pancreatic cyst diagnosis and management guidelines. The development of a standardized cyst size measurement protocol may become useful to guarantee universal reliability in pancreatic cyst management.

## Figures and Tables

**Table 1:** Summary of available pancreatic cyst management guidelines and their characterizations of high-risk pancreatic cysts.

Society		High-Risk Features That May Prompt Surgical Referral
American College of Gastroenterology (ACG) guidelines [15]	High-risk features	Tumor-related jaundice Acute pancreatitis Elevated CA 19-9 with no benign cause present Mural nodule or solid component Main pancreatic duct (PD) > 5 mm Change in main PD caliber with upstream atrophy Size > 3 cm Increase in size > 3 mm/year High-grade dysplasia or invasive malignancy
American College of Radiology (ACR) guidelines [14]	Worrisome features	Cyst diameter ≥ 3 cm Thickened or enhancing cyst wall Non-enhancing mural nodule Main PD ≥ 7 mm
	High-risk stigmata	Tumor-related jaundice Enhancing mural nodule Main PD ≥ 10 mm without obstruction Cytology with high-grade dysplasia or invasive malignancy
American Gastrointestinal Association (AGA) guidelines [13]	Positive features	Size ≥ 3 cm Dilated main PD Solid component Concerning cytology
European evidence-based guidelines [16]	Absolute indications	Cytology with high-grade dysplasia or invasive malignancy Solid mass Tumor-related jaundice Enhancing mural nodule ≥ 5 mm Main PD ≥ 10 mm
	Relative indications	Growth rate ≥ 5 mm/year CA 19-9 ≥ 37 U/mL Main PD 5–9.9 mm Cyst diameter ≥ 4 cm New-onset diabetes or acute pancreatitis Enhancing mural nodule < 5 mm
International Association of Pancreatology (IAP)/Fukuoka guidelines [9]	Worrisome features	Cyst diameter ≥ 3 cm Thickened or enhancing cyst wall Enhancing mural nodule < 5 mm Main PD 5–6 mm Lymphadenopathy Abrupt change in caliber of PD with distal pancreatic atrophy Cyst growth rate ≥ 5 mm/2 years Elevated CA 19-9
	High-risk stigmata	Enhancing mural nodule > 5 mm Main PD ≥ 10 mm Tumor-related jaundice

## Data Availability

No new data were created or analyzed in this study. Data sharing is not applicable to this article.
